# Berberine in the treatment of radiation-induced skin injury: insights from proteomics and network pharmacology

**DOI:** 10.3389/fphar.2025.1542851

**Published:** 2025-05-12

**Authors:** Jie Zou, Xia-Juan Xiao, Ping Zhang, Xing-Zi Huang, Jing Wang, Chun-Qin Tao, Xiao-Lan Ou, Gong Chen, Ting-Hao He, Long Yang, Biao Huang, Dao-Jiang Yu, Yu Zhao

**Affiliations:** ^1^ Department of Vascular Surgery, The First Affiliated Hospital of Chongqing Medical University, Chongqing Medical University, Chongqing, China; ^2^ The First Affiliated Hospital of Chengdu Medical College, Chengdu, China; ^3^ Department of Plastic and Burn Surgery, The Second Affiliated Hospital of Chengdu Medical College, Nuclear Industry 416 Hospital, Chengdu, China; ^4^ Chengdu Medical College, Chengdu, China; ^5^ Department of Burn and Plastic Surgery, Affiliated Hospital of Jiangnan University, Wuxi, China

**Keywords:** berberine, radiation-induced skin injury (RISI), proteomics, network pharmacology, wound healing

## Abstract

**Background:**

Radiation-induced skin injury (RISI) is a notable complication of cancer radiotherapy, impacting patients’ quality of life. Existing interventions mainly address symptoms, with limited success in targeting the fundamental mechanisms. Berberine (BBR), a bioactive compound recognized for its anti-inflammatory, antioxidant, and anti-fibrotic characteristics, presents a compelling option for treating RISI.

**Methods:**

The molecular targets of BBR and RISI were identified using Swiss Target Prediction and GeneCards databases. A protein-protein interaction (PPI) network was then constructed, and core targets were screened with the Cytoscape plug-in. Molecular functions and pathways were analyzed through GO and KEGG pathway enrichment analyses. Proteomic analysis identified differential protein expression following BBR treatment. Molecular docking validated BBR’s binding to core targets PRKACA and PIK3CB. Finally, the therapeutic efficacy of BBR was confirmed in irradiated cell and animal models.

**Results:**

BBR is pivotal in modulating molecular pathways linked to inflammation, oxidative stress, and tissue repair. Protein histology indicates a marked increase in epithelial migration and proliferation markers (KRT14, KRT16) and a decrease in inflammatory markers (IL6ST, TNFRSF10B). Enrichment of pathways like the MAPK cascade and epithelial development highlights BBR’s role in skin regeneration. Molecular docking confirms BBR’s stable binding to key targets PRKACA and PIK3CB, essential for cell proliferation and inflammation control. Moreover, BBR treatment promoted the proliferation of irradiated cells and accelerated wound healing in irradiated animal models.

**Conclusion:**

Berberine demonstrates multi-target therapeutic potential in managing RISI by modulating inflammation, oxidative stress, and cellular repair processes. These findings provide a foundation for future clinical studies to optimize its dosage and delivery, aiming to improve treatment outcomes for RISI.

## Introduction

Radiation therapy is a cornerstone of oncology, effectively targeting malignant cells (Liangliang et al., 2024). However, it often leads to radiation-induced skin injury (RISI), affecting 85%–95% of patients (Yan et al., 2024). RISI manifests as erythema (Abdlaty, 2016), xerosis, severe desquamation, ulceration, and late-stage fibrosis, significantly impairing quality of life and causing treatment interruptions in 10%–15% of cases (Yiren et al., 2024). The pathophysiology involves DNA strand breaks, ROS-induced oxidative stress, activation of pro-inflammatory cytokines (notably TGF-β and IL-6), and dysregulated fibroblast proliferation (Tao et al., 2024). Current treatments, such as topical corticosteroids, hydrogel dressings, and barrier creams, offer only palliative relief and do not effectively prevent chronic radiation dermatitis (Deng et al., 2024). This highlights the urgent need for targeted interventions that address the molecular mechanisms of RISI while promoting tissue regeneration.

Berberine (BBR), a natural isoquinoline alkaloid found in Berberis species and other traditional medicinal plants, has gained attention as a potential therapeutic agent for RISI management due to its diverse mechanisms of action (Amin et al., 2023). Historically used in traditional medicine to treat gastrointestinal disorders, diabetes, and infections, berberine has shown radioprotective properties against radiation-induced organ damage in recent studies (Xiao-Yu et al., 2024). Experimental models indicate its ability to lower the incidence of radiation pneumonitis, improve pulmonary oxygenation, and downregulate fibrogenic markers such as TGF-β1 in irradiated lung tissue (Yunfang et al., 2008). By targeting oxidative stress, inflammatory signaling, and tissue repair processes, berberine offers a promising multimodal approach for managing RISI.

This study utilized network pharmacology to explore the therapeutic mechanisms and key targets of BBR in managing RISI. Quantitative proteomic analysis demonstrated BBR’s influence on protein expression in irradiated cells, pinpointing two core targets validated via molecular docking (binding energies ≤−7.8 kcal/mol). BBR treatment significantly improved cell proliferation and reduced cell death in irradiated cells. Additionally, oral administration of BBR (50 mg/kg) in an animal model significantly mitigated RISI progression, underscoring BBR’s potential as a multifunctional phytotherapeutic agent for RISI prevention and treatment.

## Materials and methods

### Target prediction of BBR

The molecular structure of BBR was obtained from the PubChem database (https://pubchem.ncbi.nlm.nih.gov/) (Sunghwan et al., 2022). The compound then submitted to the Swiss Target Prediction platform (http://www.swisstargetprediction.ch/) for potential target screening (Antoine et al., 2019), with a probability threshold set at >0. Using the UniProt database (https://www.uniprot.org/), we systematically mapped the corresponding protein identifiers to their respective gene names (UniProt: the Universal Protein Knowledgebase). This integrated bioinformatics approach successfully identified a comprehensive set of potential molecular targets for BBR.

### RISI target prediction

The target genes associated with radiation-induced skin injury (RISI) were retrieved from the GeneCards database (https://www.genecards.org/) using the search term “radiation-induced skin injury” (Gil et al., 2016). After consolidating the identified targets, their gene symbols were standardized using the UniProt database. These RISI-related targets were then cross-referenced with the potential therapeutic targets of berberine (BBR). The overlapping genes, which may represent BBR’s mechanistic targets against RISI, were visualized using a Venn diagram for further functional analysis.

### Protein–protein interaction network construction

The intersecting target genes were analyzed using the STRING database (version 11.5; http://string-db.org/) with the species limited to *Homo sapiens* and an interaction confidence score threshold of <0.4 (Damian et al., 2022). The resulting protein-protein interaction (PPI) network was exported in TSV format and visualized using Cytoscape 3.9.0 for topological analysis (Paul et al., 2003). Densely interconnected network modules were identified with the MCODE plugin (v2.0.0), which implements a graph-theoretical clustering algorithm specifically optimized for biological networks. Nodes with the highest network centrality (degree centrality >15) were designated as hub targets for further functional validation.

### GO and KEGG pathway enrichment analysis

Functional annotation and pathway enrichment analyses were performed using the clusterProfiler R package (version 3.14.3) to elucidate the biological processes and signaling pathways associated with BBR’s therapeutic targets in RISI. Statistical significance thresholds were set at *p* < 0.01 with a false discovery rate (FDR) < 0.05 (The Gene Ontology Consortium, 2018). For Gene Ontology (GO) annotation, GO IDs were extracted from the identified proteins using eggnog-mapper software based on the EggNOG database (Jaime et al., 2018).

### Cell processing and protein extraction

Human umbilical vein endothelial cells (HUVECs) were cultured according to a previously described protocol (Bahare et al., 2014). The cells were seeded in flat-bottom 6-well plates and allowed to grow until they reached 80%–90% confluence using an optical microscope and an eyepiece graticule. The culture medium was then replaced with fresh medium. After an additional 24 h of cultivation, the cells were exposed to 5 Gy of 160 kVp X-ray irradiation using a RADSOURCERS 2000 X-ray machine (United States) at a dose rate of 1.20 Gy/min. Concurrently or separately, the cells were treated with 5 µM berberine (BBR) and incubated at 37.8°C in a 5% CO_2_ atmosphere for another 24 h. The sample was grinded with liquid nitrogen into cell powder and then transferred to a 5-mL centrifuge tube. After that, four volumes of lysis buffer (8 M urea, 1% protease inhibitor cocktail) were added to the cell powder, followed by sonication 3 minutes on ice using a high intensity ultrasonic processor (Scientz) (Note: For PTM experiments, inhibitors were also added to the lysis buffer, e.g., 3 μM TSA and 50 mM NAM for acetylation, 1% phosphatase inhibitor for phosphorylation). The remaining debris was removed by centrifugation at 12,000 g at 4°C for 10 min. Finally, the supernatant was collected and the protein concentration was determined with BCA kit according to the manufacturer’s instructions.

The ratio of the relative quantitative values of proteins in two samples was used as the fold change (FC). The fold change of proteins between sample A and sample B was calculated. A fold change greater than 1.5 was set as the threshold for significant upregulation, while a fold change less than 1/1.5 was set as the threshold for significant downregulation.

For the CCK-8 assay, HUVECs were seeded in 96-well plates at a density of 1.5 × 10^3^ cells/cm^2^ and divided into the following groups: a control group and BBR-treated groups with different concentrations (5 μM, 10 μM, and 20 μM). After 24 h of culture, cell proliferation was assessed using the CCK-8 kit, and the optical density (OD) at 450 nm was measured with a Bio-Rad 450 microplate reader. To evaluate the effects of BBR on irradiated cells, the same experimental procedure was repeated under 20 Gy irradiation. Additionally, cytotoxicity was determined by measuring LDH (Lactate Dehydrogenase) release in HUVECs treated with the aforementioned concentrations of BBR (5 μM, 10 μM, and 20 μM).

### Irradiated animal model and normal wound data

To investigate single-cell transcriptional changes in irradiated skin, rats were anesthetized via intraperitoneal injection of ketamine (75 mg/kg) and medetomidine (10 mg/kg), followed by shaving of the gluteal hair using a razor. A 3-cm-thick lead shield was positioned to delineate a 3 cm × 4 cm radiation field. Radiation was delivered using a 6-MeV electron beam accelerator at a dose rate of 1,000 cGy/min, with a total dose of 30 Gy administered to the target area. Skin tissues were harvested from irradiated regions at 7, 14, 28, and 60 days post-exposure (Xiaowu et al., 2019), with non-irradiated skin samples serving as controls. The resulting sequencing data have been deposited in the GEO database under accession number GSE193564 (Fenghao et al., 2024).

To further evaluate the therapeutic effects of berberine (BBR) on radiation-induced skin injuries, we established an irradiated murine model. Mice in the BBR treatment group received intragastric administration of BBR (50 mg/kg/day) for three consecutive days prior to irradiation. The radiation-induced skin ulcer model was established as previously described (Sanath et al., 2008). Briefly, mice were anesthetized with 1% pentobarbital sodium (30 mg/kg), after which their hind limbs were exposed to 160 kVp X-ray irradiation (RADSOURCERS 2000 X-ray machine, United States) at a dose rate of 1.20 Gy/min for 8–9 min, delivering a total dose of 20 Gy (Precision X-ray). Lead shielding was used to protect other body parts. Skin wound healing was monitored for 24 days post-irradiation in both BBR-treated and irradiation-only (IR) groups, with photographic documentation of wound sites. Skin injury severity was graded according to a five-point scale previously established by our research group (Rhonda et al., 2016).

Furthermore, transcriptomic data from normal wound healing at day 1, day 7, and chronic ulcer wounds were obtained from the GEO database (accession number GSE174661) (Zhuang et al., 2022). By integrating these datasets with transcriptomic data from irradiated animal skin, we analyzed the expression dynamics of the core genes PRKACA and PIK3CB under both irradiated and normal wound healing conditions.

### Molecular docking and molecular dynamic simulations

The 3D structures of BBR’s core targets in RISI were retrieved from the PDB database (https://www.rcsb.org/) (Damiano, 2024). We obtained the crystal structures of key targets from the PDB library, including PRKACA (PDB ID: 8X5L) and PIK3CB (UniProt ID: P42338). Using AutoDock Tools, we performed protein preparation by removing water molecules, adding hydrogen atoms, and assigning charges. Subsequently, molecular docking with BBR was conducted (Garrett et al., 2008). For molecular dynamics analysis, the IMOD server (http://imods.chaconlab.org) was employed to assess the stability and molecular mobility of the BBR-core target complexes. The IMODS platform additionally enabled structural dynamics analysis of the docking complexes. Elastic networks, deformability, B-factors, eigenvalues, variance, and covariance maps were all used to illustrate the stability of the BBR-core target complex.

### Statistical analysis

For comparisons between two groups, Student’s t-test or Mann-Whitney U test was applied based on data distribution. One-way analysis of variance (ANOVA) followed by Tukey’s *post hoc* test was used for multiple group comparisons. Two-way ANOVA was employed to analyze the interaction effects between different treatment conditions. In all tests, a *p*-value <0.05 or a false discovery rate (FDR) < 0.05 was considered the threshold for statistical significance.

## Results

### Targets of BBR and RISI

**FIGURE 1:**
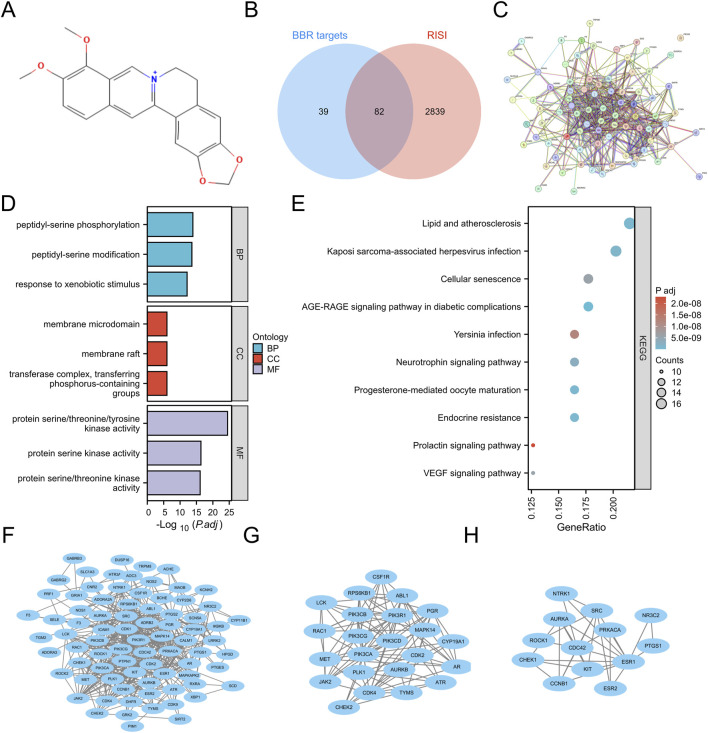
Structure and potential targets of BBR. **(A)** Structure of BBR. **(B)** Overlapping targets between RISI and BBR. **(C)** PPI network of overlapping targets. **(D,E)** GO and KEGG pathway enrichment analysis. **(F–H)** Construction of PPI network, subnetwork 1 and subnetwork 2 via Cytoscape.

The potential targets of BBR were explored by inputting its chemical structure (Figure 1A) in SMILES format. An initial examination identified 121 potential BBR-associated targets, offering initial understanding of its mechanism of action. Subsequently, 6,880 associated targets were discovered according to the GeneCards database. After integrating the data and applying stringent threshold-based filtering, a final cohort of 2,921 RISI-related target genes was defined for subsequent analysis (Figure 1B). Through Venn diagram analysis, we identified 82 overlapping targets. These candidate targets were prioritized based on their dual involvement as both molecular targets of berberine and genetic markers associated with RISI (Figure 1B).

### GO and KEGG results

Functional enrichment analysis (GO/KEGG) of the 82 overlapping targets (FDR <0.05) revealed 1,838 significant terms. The analysis encompassed 1,644 biological processes, including peptidyl-serine phosphorylation, xenobiotic metabolic response, cell cycle regulation (particularly G2/M transition), and MAPK cascade activation. Cellular component analysis revealed 71 terms, primarily involving membrane structures (rafts, microdomains, caveolae) and kinase complexes (PI3K, cyclin-dependent, serine/threonine). Molecular function analysis identified 123 terms, predominantly related to kinase activities (serine/threonine/tyrosine, histone), binding functions (heme, tetrapyrrole, insulin receptor substrate) and enzymatic activities (PI3K, PIP2 kinase) (Figure 1D). KEGG pathway enrichment analysis identified 161 significant pathways (Figure 1E), including the AGE-RAGE signaling pathway in diabetic complications, endocrine resistance, progesterone-mediated oocyte maturation, lipid metabolism and atherosclerosis, Kaposi sarcoma-associated herpesvirus infection, neurotrophin signaling pathway, VEGF signaling pathway, cellular senescence, *Yersinia* infection, and prolactin signaling pathway.

### PPI network construction and hub targets selection

Subsequent STRING database analysis demonstrated significant functional interactions among proteins encoded by the 82 overlapping targets (Figure 1C). The network contained 81 nodes and 488 edges (Figure 1F). MCODE plugin analysis identified two functionally significant subnetworks: Subnetwork 1 (23 nodes, 105 edges) contained core targets including ATR, CDK4, ABL1, JAK2, RAC1, PIK3CB, PIK3CA, AR, PLK1, CDK2, AURKB, MET, CSF1R, CYP19A1, PGR, PIK3CD, PIK3CG, RPS6KB1, LCK, TYMS, PIK3R1, MAPK14, and CHEK2 (Figure 1G), while Subnetwork 2 (13 nodes, 30 edges) featured key targets such as NTRK1, CHEK1, AURKA, PRKACA, ESR1, ROCK1, SRC, KIT, CDC42, ESR2, CCNB1, PTGS1, and NR3C2 (Figure 1H). Targets from both subnetworks were prioritized as hub targets for subsequent mechanistic investigations.

### Proteomics analysis and subcellular localization

**FIGURE 2:**
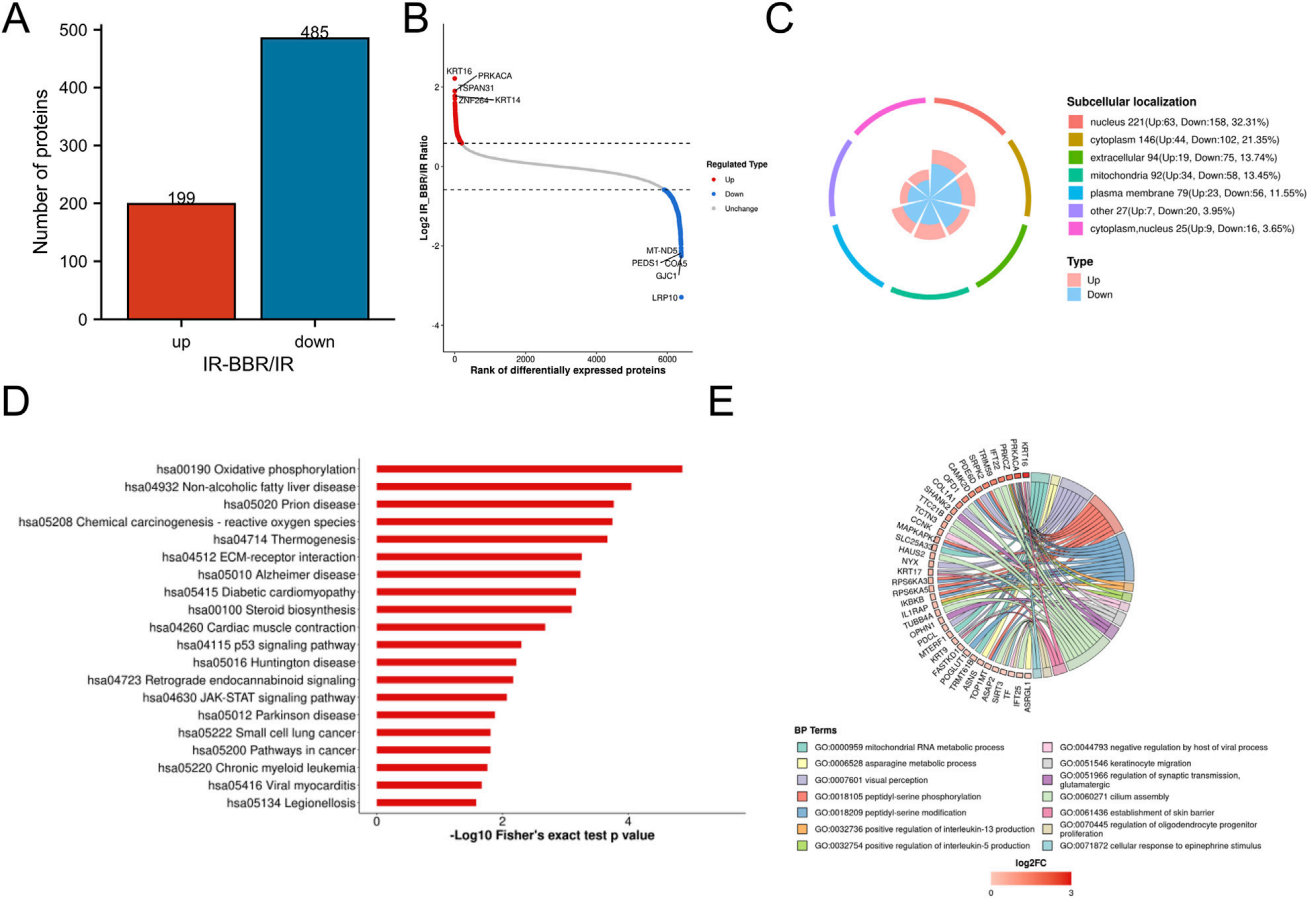
Proteomics analysis of BBR treatment. **(A)** Statistical analysis of differential proteins. **(B)** Top differential proteins with significant upregulation and downregulation. **(C)** Subcellular location of differential proteins. **(D)** Top 20 Downregulated KEGG pathways by BBR. **(E)** Biological processes of proteins upregulated by BBR.

We conducted comparative proteomic analysis to characterize protein expression changes in irradiated cells with and without berberine treatment. Quantitative analysis revealed that BBR treatment significantly modulated the irradiated cell proteome, upregulating 199 proteins (KRT14, KRT16 and PRKACA) while downregulating 485 proteins (notably LRP10 and GJC1) (Figures 2A,B). Subcellular localization profiling demonstrated distribution of these differentially expressed proteins, with predominant nuclear localization (32.31%), followed by cytoplasmic (21.35%), extracellular (13.74%), mitochondrial (13.45%), and plasma membrane (11.55%) compartments (Figure 2C).

### Functional annotation of differentially expressed proteins

BBR significantly upregulated proteins involved in critical biological processes, including mitochondrial RNA metabolism, peptidyl-serine phosphorylation, keratinocyte activation, interleukin-5 (IL-5) production, skin barrier formation, and the cellular response to epinephrine (Figure 2D). Conversely, it downregulated pathways linked to inflammatory responses and radiation-induced damage, such as oxidative phosphorylation, p53 signaling, chemical carcinogenesis (reactive oxygen species, ROS), diabetic cardiomyopathy, JAK-STAT signaling, and oncogenic pathways (Figure 2E).

### Core targets selection

**FIGURE 3:**
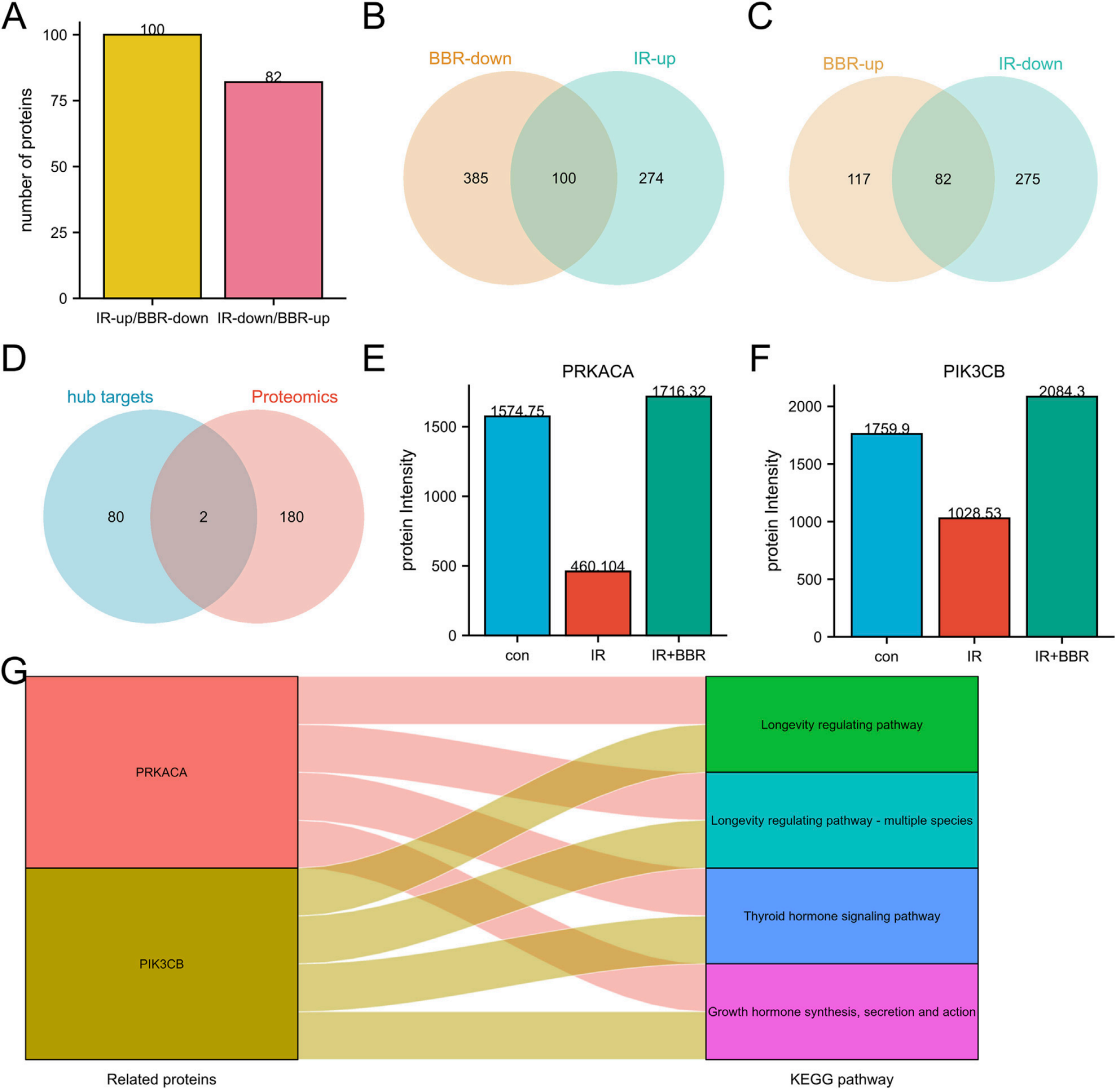
Core targets screening of BBR in RISI treatment. **(A–C)** Identification of potential targets regulated by BBR in RISI through proteomics analysis. **(D)** Intersection of proteomics targets and hub targets. **(E,F)** Protein expression differences of core targets between irradiation and BBR treatment groups. **(G)** Key pathways involved by core targets (PRKACA and PIK3CB).

Intersection analysis revealed proteins differentially regulated by irradiation and BBR treatment, including those downregulated by irradiation but upregulated by BBR, and *vice versa* (Figures 3A–C). These proteins are potential key regulatory targets of BBR. Among 182 candidate regulatory proteins, we cross-referenced 82 hub targets from network pharmacology screening with proteomics data, identifying two core targets: PRKACA and PIK3CB (Figures 3D–F). Notably, their roles in longevity regulation, growth hormone signaling, and thyroid hormone pathways highlight their critical importance in fundamental cellular processes and associated disease mechanisms (Figure 3G).

### Expression validation and co-expression analysis

**FIGURE 4:**
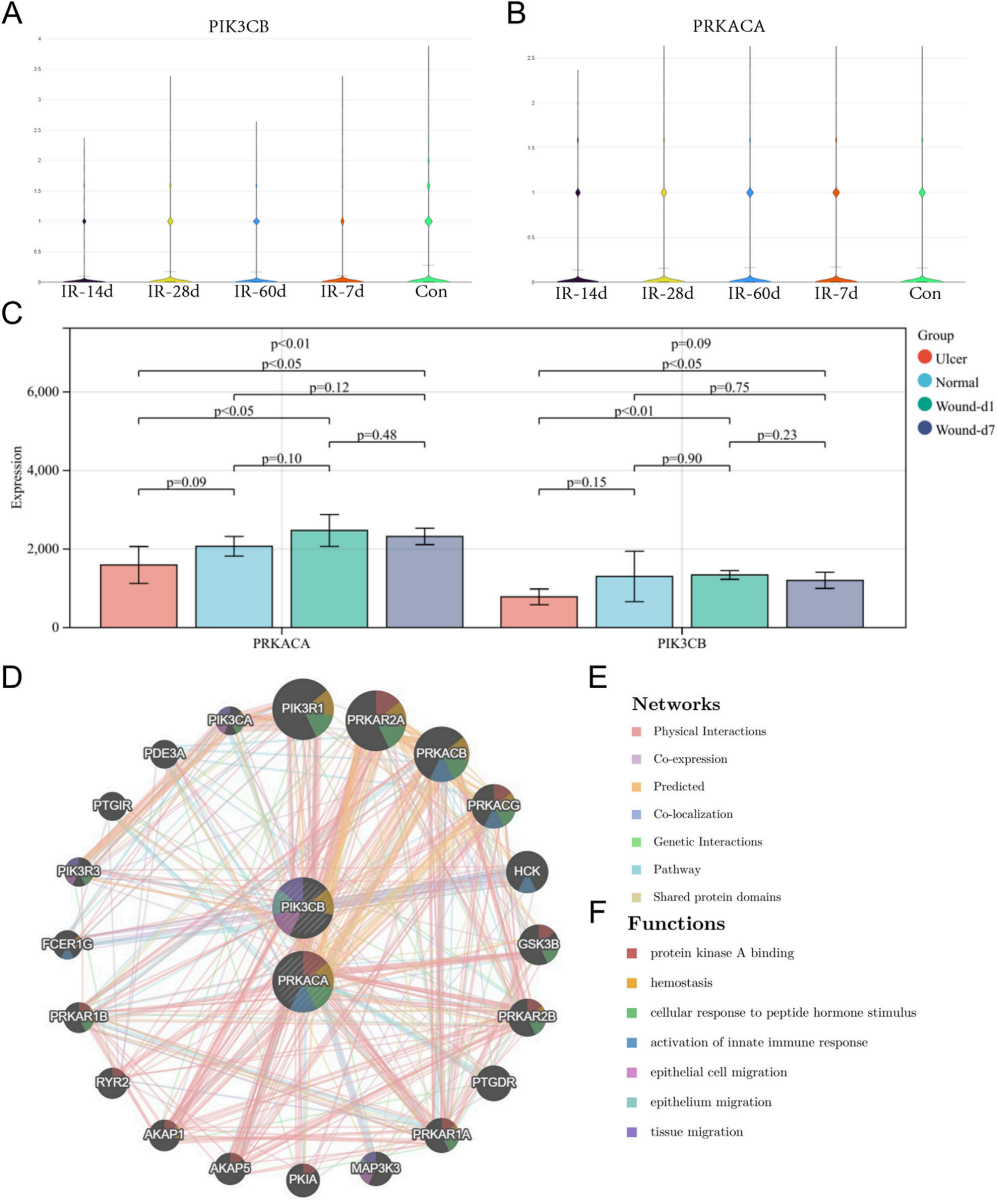
Expression validation and co-expression analysis of core targets. **(A)** Expression changes of PIK3CB in animal skin tissues at different time points (7, 14, 28, 60 days) after irradiation. **(B)** Expression changes of PRKACA in animal skin tissues after irradiation. **(C)** Expression changes of core targets during normal wound healing and in chronic ulcers. **(D)** Co-expression network and functional analysis of core targets.

In rat skin tissues, PIK3CB expression decreased after irradiation, reaching its nadir on day 14 (Figure 4A). PRKACA expression showed a similar decline by day 14 (Figure 4B). During normal wound healing, these genes exhibited a non-significant upregulation following acute tissue injury when compared to control groups (Figure 4C). Importantly, PRKACA and PIK3CB levels were significantly elevated in normal wounds compared to chronic ulcers (Figure 4C). Co-expression network analysis revealed that PRKACA, PIK3CB, and their associated genes were primarily involved in epithelial cell migration, activation of the innate immune response, tissue migration, cellular response to peptide hormone stimulation, and protein kinase A binding (Figures 4D–F).

### Molecular docking and molecular dynamic simulations results

**FIGURE 5:**
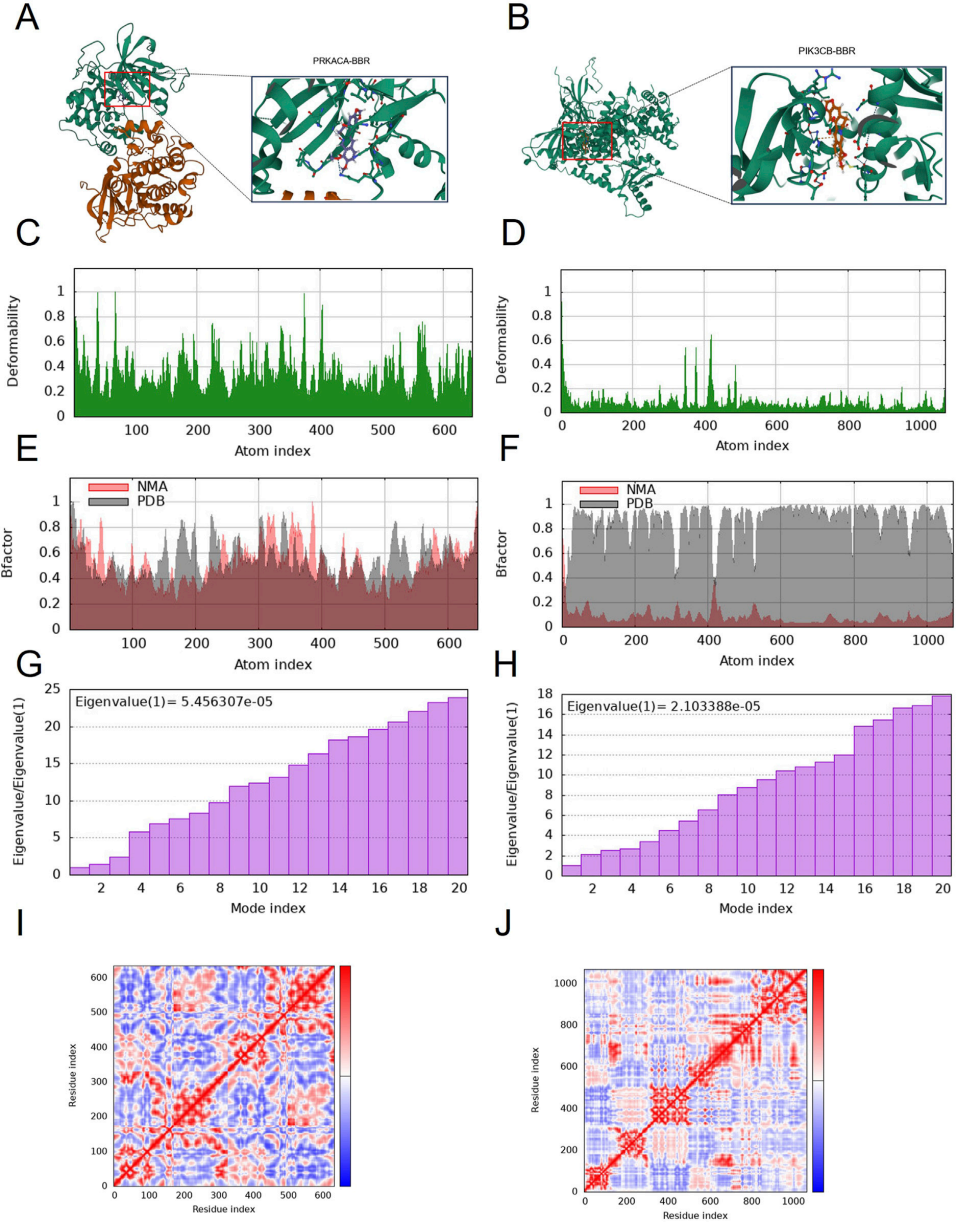
Molecular docking and dynamic simulation. **(A)** Binding mode of PIK3CB to berberine. **(B)** Binding mode of PRKACA to berberine. Three-dimensional structures of the binding pockets were showed by Pymol software. **(C–F)** deformability and B-factor plots. **(G,H)** eigenvalue plots (Colored bars show the individual (purpule). **(I,J)** covariance map.

Molecular docking simulates ligand-protein interactions, with binding energy (affinity) determining docking quality. Lower affinity values indicate stronger binding, where values < −7 kcal mol^−1^ typically suggest effective interactions. PRKACA and PIK3CB exhibited particularly stable binding, with energies ranging from −7.723 to −8.409 kcal mol^−1^ (Table 1). Figures 5A,B details the docking targets and their corresponding active compounds.

**TABLE 1:** Docking scores between component and core targets.

Protein	Ligand	Binding energy (kcal·mol^–1^)	Binding site	Applied force
PRKACA	Berberine	−7.723	ARG97	Cation-Pi interaction
PIK3CB	Berberine	−8.409	LYS169	Hydrogen bond

The peaks in Figures 5C,D indicate regions of protein deformability (backbone distortion). Protein hinges, which are not structurally significant, contribute to overall stability, while high-deformability regions correspond to chain hinge locations. In this study, the B-factor quantifies residue-specific molecular deformability (Figures 5E,F). Molecular motion stiffness is characterized by eigenvalues associated with each normal mode, with calculated values of 5.456307 × 10^−5^ and 2.103388 × 10^−5^ in this analysis (Figures 5G,H). Higher eigenvalues indicate greater variance, while lower values correspond to simpler deformations. The covariance map (Figures 5I,J) displays residue motion correlations: red represents correlated motion, blue indicates anti-correlated motion, and white shows no correlation.

### BBR promotes cell proliferation and mitigates radiation-induced damage

**FIGURE 6:**
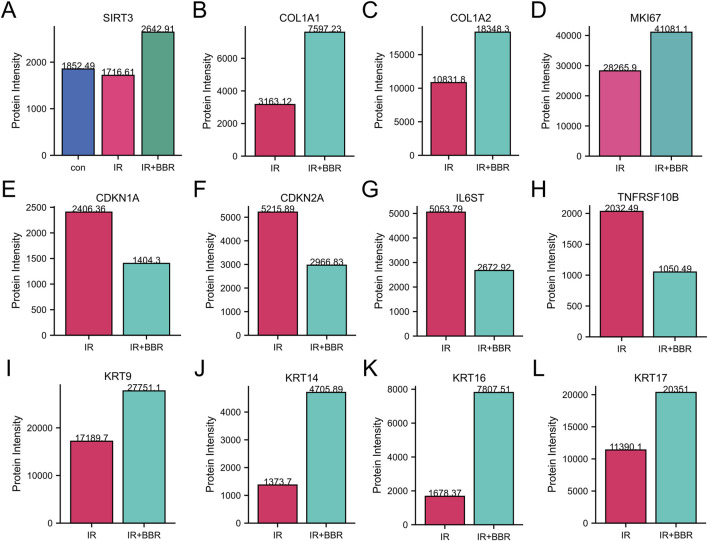
BBR regulates markers associated with cellular inflammation and proliferation. **(A)** BBR upregulated longevity-associated protein SIRT3. **(B–D)** BBR upregulated skin repair-associated protein COL1A1, COL1A2 and MKI67. **(E,F)** BBR downregulated aging-related proteins such as CDKN1A and CDKN2A. **(G,H)** BBR downregulated inflammatory markers like IL6ST and TNFRSF10B. **(I–L)** BBR upregulated key markers of keratinocyte activation and epithelial migration (KRT9, KRT14, KRT17 and KRT16).

Our analysis identified a strong link between core targets and wound healing, especially in pathways related to longevity regulation and epithelial cell migration. Protein expression analysis showed that BBR treatment increased proliferation and remodeling markers (MKI67, COL1A1, COL1A2) and epithelial migration markers (KRT9, 14/16/17), while decreasing aging-related proteins (CDKN1A, CDKN2A) and inflammatory markers (IL6ST, TNFRSF10B) (Figures 6B–L). Importantly, BBR reversed the irradiation-induced decline of the longevity marker SIRT3 (Figure 6A).

**FIGURE 7:**
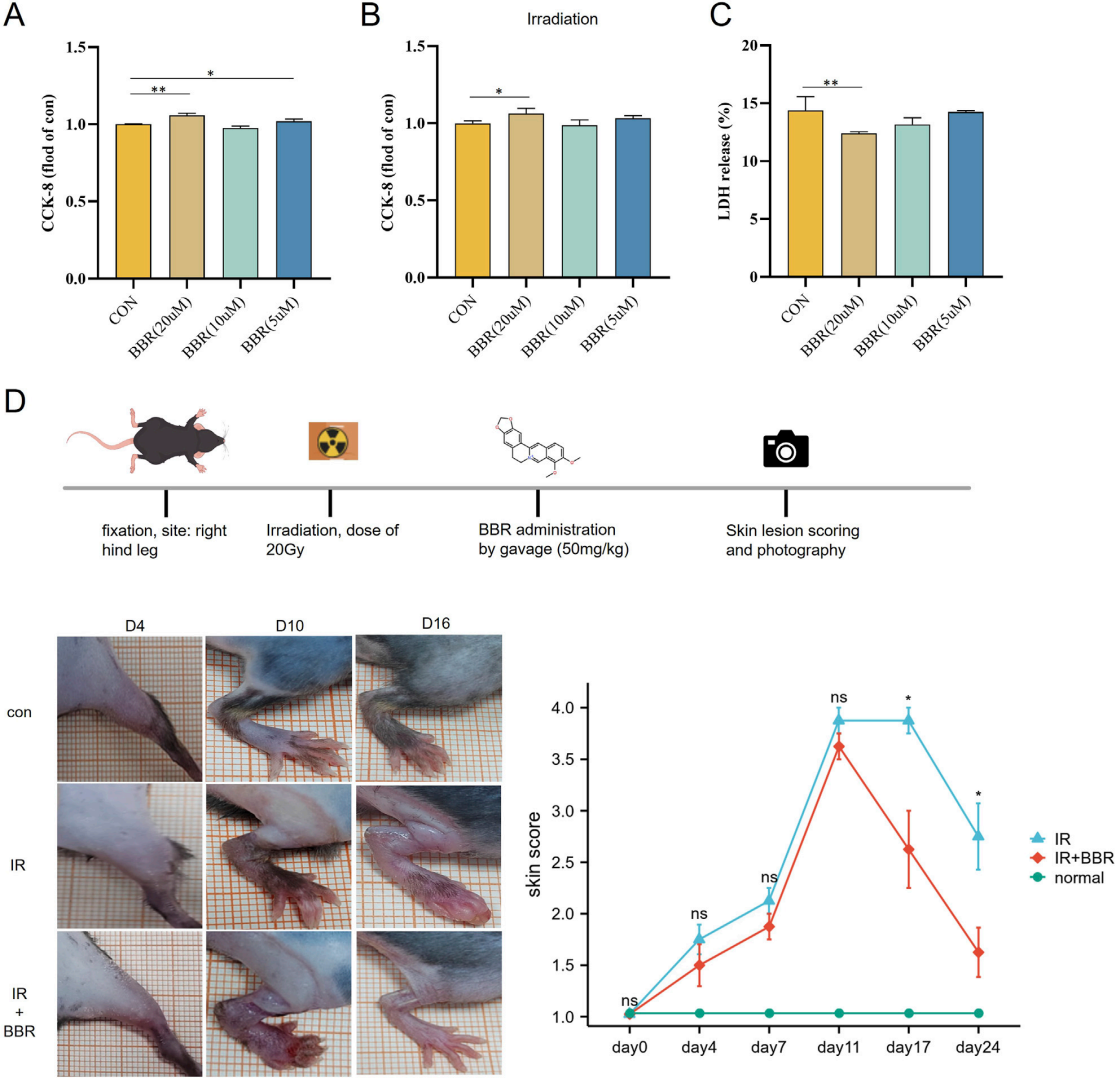
Therapeutic effect of BBR on irradiated cells and animal models. **(A)** CCK-8 were used to assess cell proliferation after BBR treatment. **(B)** CCK-8 were used to assess irradiated cell proliferation after BBR treatment. **(C)** detection of LDH level under different concentrations of BBR treatment. **(D)** pictures showing the scoring curves of the whole course of radiogenic injury in mice with different BBR treatment conditions.

CCK-8 assays assessed BBR’s impact on HUVEC proliferation, revealing that both 5 μM and 20 µM BBR significantly promoted cell proliferation in non-irradiated conditions (Figure 7A). Under irradiated conditions, 20 µM BBR notably enhanced proliferation (Figure 7B). Consistent with these results, LDH activity assays showed that 20 µM BBR significantly lowered LDH levels, indicating reduced cell death (Figure 7C).

### BBR accelerated the skin healing process in irradiated animals

After exposing the hind legs of mice to 20 Gy irradiation, we systematically tracked skin damage progression using continuous observation (two experienced observers examined the skin damage of the animal models under blinded conditions for 24 days) and photographic documentation. Skin damage scores steadily increased from day 4 to day 11 post-irradiation, then gradually declined. Comparison between the irradiation-only group and the BBR-treated group indicated that oral BBR significantly accelerated skin healing, with marked improvements noted at 17 and 24 days post-irradiation (Figure 7D).

## Discussion

Radiation-induced skin injury (RISI) is a frequent complication of radiation therapy, presenting symptoms from erythema and desquamation to severe ulceration and fibrosis (Tao et al., 2024; Patricia et al., 2024). These effects significantly impair patients’ quality of life and can interrupt crucial cancer treatments (Xiaojing et al., 2020). Current management strategies focus on symptom relief and wound healing, yet definitive curative interventions are lacking (Manni et al., 2016). This underscores the urgent need for more effective therapeutic approaches.

Our study highlights berberine’s therapeutic potential in mitigating radiation-induced skin injury (RISI). Utilizing network pharmacology, proteomics, animal models, and molecular docking analyses, we demonstrate that berberine effectively targets key molecular pathways and proteins linked to inflammation, oxidative stress, and tissue repair. These results provide a detailed understanding of berberine’s promise as a therapeutic agent for RISI.

Proteomic analysis identified notable changes in proteins related to skin repair, inflammation regulation, and cellular aging. Berberine specifically upregulated key markers of keratinocyte activation and epithelial migration, such as KRT14 and KRT16, while downregulating inflammatory markers like IL6ST and TNFRSF10B (Xiaowei et al., 2019; Minseok et al., 2024). This dual action indicates that berberine mitigates radiation-induced inflammation while promoting skin regeneration and enhancing barrier function.

GO and KEGG pathway enrichment analyses elucidated the molecular mechanisms of berberine’s effects. Significant enrichment was observed in biological processes such as MAPK cascade regulation, epithelial cell development, and the G2/M transition of the mitotic cell cycle, highlighting berberine’s potential in cell cycle progression and tissue regeneration (Maryam et al., 2025). Additionally, downregulated pathways like oxidative phosphorylation and p53 signaling correspond with reduced oxidative stress and DNA damage following berberine treatment (Zaigang et al., 2024; Anh Phong et al., 2022).

Our findings highlight berberine’s potential to modulate aging-related pathways. The marked downregulation of senescence-associated proteins, such as CDKN1A and CDKN2A, suggests berberine’s efficacy in mitigating radiation-induced aging in skin cells (Zhangyang et al., 2024; Grace et al., 2024). Moreover, the upregulation of SIRT3, a key protein associated with longevity, supports berberine’s role in enhancing cellular resilience against radiation-induced damage (Wei et al., 2024; Zhang et al., 2024).

The identification of PRKACA and PIK3CB as key targets underscores berberine’s molecular specificity in modulating vital biological processes. Both targets are integral to pathways involved in cellular repair, proliferation, and inflammation (Youyi et al., 2024; Wencui et al., 2024). Molecular docking and dynamics analyses confirmed strong binding affinities between berberine and these targets, suggesting stable interactions that likely drive its therapeutic effects. In irradiated animal models, downregulation of PRKACA and PIK3CB expression was noted, correlating with the development of chronic ulcers during wound healing. Thus, BBR has the potential to ameliorate radiation-induced damage by upregulating the expression of these key targets. The co-expression network of PRKACA and PIK3CB underscores their involvement in pathways related to epithelial migration, innate immune activation, and peptide hormone signaling (Xiong et al., 2024). These interactions highlight berberine’s complex role in modulating local skin repair and systemic responses to radiation-induced injury.

Finally, the *in vivo* and *in vitro* effects of different concentrations of BBR were validated. We developed irradiated animal and cell models to examine BBR’s therapeutic effects on radiation-induced injuries. Our results indicate that BBR significantly boosts the proliferation of irradiated cells and speeds up skin damage recovery in irradiated animals. These findings suggest BBR as a promising candidate for the clinical prevention and treatment of radiation injuries.

Despite these promising results, several limitations warrant acknowledgment. First, the *in vivo* relevance of the identified targets and pathways requires further validation through animal models and clinical trials. Second, the precise dosage and delivery mechanisms for berberine in the context of RISI need optimization.

In conclusion, this study identifies berberine as a multi-targeted agent with significant potential for managing RISI. By modulating inflammation, oxidative stress, and cellular repair pathways, berberine addresses both the symptoms and underlying causes of radiation-induced skin damage. Future research should focus on translating these findings into clinical applications, potentially offering cancer patients a more effective and comprehensive treatment for radiation-related skin injuries.

## Data Availability

The data supporting this publication are available in NCBI’s Gene Expression Omnibus (GEO) under accession numbers GSE193564 and GSE174661.

## References

[B1] Abdlaty R. (2016). Hyperspectral imaging and data analysis of skin erythema post radiation therapy treatment. McMaster university. Available online at: https://macsphere.mcmaster.ca/handle/11375/20765

[B2] Amin G. Farah A. Saba Z. Petro O. Oksana K. Roman L. (2023). Berberine: pharmacological features in health, disease and aging. Curr Med Chem. 31 (10), 0. 10.2174/0929867330666230207112539 36748808

[B3] Anh Phong T. Tralie C. J. Reyes J. Moosmüller C. Belkhatir Z. Kevrekidis I. G. (2022). Long-term p21 and p53 dynamics regulate the frequency of mitosis events and cell cycle arrest following radiation damage. Cell Death Differ. 30 (3), 660–672. 10.1038/s41418-022-01069-x 36182991 PMC9984379

[B4] Antoine D. Olivier M. Vincent Z. (2019). SwissTargetPrediction: updated data and new features for efficient prediction of protein targets of small molecules. Nucleic Acids Res. 47 (0), W357–W364. 10.1093/nar/gkz382 31106366 PMC6602486

[B5] Bahare F. Rafatpanah H. Ravari H. Farid Hosseini R. Tavakol Afshari J. Hamidi Alamdari D. (2014). Sera of patients with thromboangiitis obliterans activated cultured human umbilical vein endothelial cells (HUVECs) and changed their adhesive properties. Int. J. Rheum. Dis. 17 (1), 106–112. 10.1111/1756-185X.12214 24472273

[B6] Damian S. Kirsch R. Koutrouli M. Nastou K. Mehryary F. Hachilif R. (2022). The STRING database in 2023: protein-protein association networks and functional enrichment analyses for any sequenced genome of interest. Nucleic Acids Res. 51 (0), D638–D646. 10.1093/nar/gkac1000 PMC9825434 36370105

[B7] Damiano C. (2024). RepeatsDB in 2025: expanding annotations of structured tandem repeats proteins on AlphaFoldDB. Nucleic Acids Res. 10.1093/nar/gkae965 PMC11701623 39475178

[B8] Deng G. Weishan H. Wenya L. Fasheng W. Jibing C. (2024). Evolution of radiation-induced dermatitis treatment. Clin. Transl. Oncol. 26 (9), 2142–2155. 10.1007/s12094-024-03460-1 38594379

[B9] Fenghao G. Zhong L. Yang T. Chen J. Yang P. Jiang F. (2024). A frog skin-derived peptide targeting SCD1 exerts radioprotective effects against skin injury by inhibiting STING-mediated inflammation. Adv. Sci. (Weinh) 11 (25), e2306253. 10.1002/advs.202306253 38582510 PMC11220654

[B10] Garrett M. M. Ruth H. Arthur J O. (2008). Using AutoDock for ligand-receptor docking. Curr. Protoc. Bioinforma. 8, 8.14. 10.1002/0471250953.bi0814s24 19085980

[B11] Gil S. Rosen N. Plaschkes I. Zimmerman S. Twik M. Fishilevich S. (2016). The GeneCards suite: from gene data mining to disease genome sequence analyses. Curr. Protoc. Bioinforma. 54 (0), 1.30.1–1.30.33. 10.1002/cpbi.5 27322403

[B12] Grace T. Y. Paul T. G. Larissa G. P. Julia S. L. Tamar T. James L. K. (2024). Clinicopathological and cellular senescence biomarkers in chronic stalled wounds. Int. J. Dermatol 63 (9), 1227–1235. 10.1111/ijd.17072 38351588 PMC11323232

[B13] Jaime H.-C. Szklarczyk D. Heller D. Hernández-Plaza A. Forslund S. K. Cook H. (2018). eggNOG 5.0: a hierarchical, functionally and phylogenetically annotated orthology resource based on 5090 organisms and 2502 viruses. Nucleic Acids Res. 47 (0), D309–D314. 10.1093/nar/gky1085 PMC6324079 30418610

[B14] Liangliang W. Lynch C. Pitroda S. P. Piffkó A. Yang K. Huser A. K. (2024). Radiotherapy and immunology. J. Exp. Med. 221 (7). 10.1084/jem.20232101 PMC11110906 38771260

[B15] Manni S. Alavi A. Wong R. Akita S. (2016). Radiodermatitis: a review of our current understanding. Am. J. Clin. Dermatol 17 (3), 277–292. 10.1007/s40257-016-0186-4 27021652

[B16] Maryam K. Nouri F. Yousefi M. H. Pajouhi A. Ghorbani H. Afkhami H. (2025). Mesenchymal stem cells and their exosomes: a novel approach to skin regeneration via signaling pathways activation. J. Mol. Histol. 56 (2), 132. 10.1007/s10735-025-10394-7 40208456

[B17] Minseok K. Kim C. Zheng H. Kim Y. Cho P. S. Lim J. Y. (2024). Pharmacologic inhibition of Il6st/gp130 improves dermatological inflammation and pruritus. Biomed. Pharmacother. 178 (0), 117155. 10.1016/j.biopha.2024.117155 39047422

[B18] Patricia F. B. Vidya P. K. Yali K. Kan W. Howard L. David M. (2024). Radiation induced skin fibrosis (RISF): opportunity for angiotensin II-dependent intervention. Int. J. Mol. Sci. 25 (15), 8261. 10.3390/ijms25158261 39125831 PMC11312688

[B19] Paul S. Markiel A. Ozier O. Baliga N. S. Wang J. T. Ramage D. (2003). Cytoscape: a software environment for integrated models of biomolecular interaction networks. Genome Res. 13 (11), 2498–2504. 10.1101/gr.1239303 14597658 PMC403769

[B20] Rhonda M. B. Michael W, E. Mark J. S. Erin M, S. Xiang G. Song L. (2016). A topical mitochondria-targeted redox-cycling nitroxide mitigates oxidative stress-induced skin damage. J Invest Dermatol. 137 (3), 0. 10.1016/j.jid.2016.09.033 PMC5466072 27794421

[B21] Sanath K. Kolozsvary A. Kohl R. Lu M. Brown S. Kim J. H. (2008). Radiation-induced skin injury in the animal model of scleroderma: implications for post-radiotherapy fibrosis. Radiat. Oncol. 3 (0), 40. 10.1186/1748-717X-3-40 19025617 PMC2599892

[B22] Sunghwan K. Chen J. Cheng T. Gindulyte A. He J. He S. (2022). PubChem 2023 update. Nucleic Acids Res. 51 (0), D1373–D1380. 10.1093/nar/gkac956 PMC9825602 36305812

[B23] Tao Y. Yang P. Bai H. Song B. Liu Y. Wang J. (2024). Single-cell RNA-Seq analysis of molecular changes during radiation-induced skin injury: the involvement of Nur77. Theranostics 14 (15), 5809–5825. 10.7150/thno.100417 39346541 PMC11426238

[B24] The Gene Ontology Consortium (2018). The gene Ontology resource: 20 years and still GOing strong. Nucleic Acids Res. 47 (0), D330–D338. 10.1093/nar/gky1055 PMC6323945 30395331

[B25] UniProt Consortium (2025). UniProt: the universal protein knowledgebase in 2025 *.* Nucleic Acids Res. 53 (0), 0. 10.1093/nar/gkae1010 PMC11701636 39552041

[B26] Wei W. Li T. Chen J. Fan Z. Gao F. Yu Z. (2024). SIRT3/6: an amazing challenge and opportunity in the fight against fibrosis and aging. Cell. Mol. life Sci. CMLS 81 (1), 69. 10.1007/s00018-023-05093-z 38294557 PMC10830597

[B27] Wencui K. Feng X. Yu Z. Qi X. Zhao Z. (2024). “USP8-mediated PTK7 promotes PIK3CB-related pathway to accelerate the malignant progression of non-small cell lung cancer.” Thorac. Cancer. 16. 10.1111/1759-7714.15485 PMC11729734 39552193

[B28] Xiaojing Y. Ren H. Guo X. Hu C. Fu J. (2020). Radiation-induced skin injury: pathogenesis, treatment, and management. Aging (Albany NY) 12 (22), 23379–23393. 10.18632/aging.103932 33202382 PMC7746368

[B29] Xiaowei Z. Meimei Y. Ling-Juan Z. (2019). Keratin 6, 16 and 17-critical barrier alarmin molecules in skin wounds and psoriasis. Cells 8 (8), 807. 10.3390/cells8080807 31374826 PMC6721482

[B30] Xiaowu S. Zhou Y. Wang H. Shen Y. Liao Q. Rao Z. (2019). Establishment and characterization of a radiation-induced dermatitis rat model. J. Cell Mol. Med. 23 (5), 3178–3189. 10.1111/jcmm.14174 30821089 PMC6484338

[B31] Xiao-Yu Z. Yu L. Wang K. Wang M. Li P. Zheng Z. G. (2024). The combination of berberine and isoliquiritigenin synergistically improved adipose inflammation and obesity-induced insulin resistance. Phytother. Res. 38 (8), 3839–3855. 10.1002/ptr.8233 38729776

[B32] Xiong L. Zhevlakova I. West X. Z. Gao D. Murtazina R. Horak A. (2024). TLR2 regulates hair follicle cycle and regeneration via BMP signaling. eLife 12. 10.7554/eLife.89335 PMC10939499 38483447

[B33] Yan C. Ma L. Cheng Z. Hu Z. Xu Y. Wu J. (2024). Senescent fibroblast facilitates re-epithelization and collagen deposition in radiation-induced skin injury through IL-33-mediated macrophage polarization. J. Transl. Med. 22 (1), 176. 10.1186/s12967-024-04972-8 38369466 PMC10874572

[B34] Yiren W. Chen S. Bao S. Yao L. Wen Z. Xu L. (2024). Deciphering the fibrotic process: mechanism of chronic radiation skin injury fibrosis. Front. Immunol. 15 (0), 1338922. 10.3389/fimmu.2024.1338922 38426100 PMC10902513

[B35] Youyi L. Wang B. Cheng Y. Fang Y. Hou Y. Mao Y. (2024). ASIC1 promotes migration and invasion of hepatocellular carcinoma via the PRKACA/AP-1 signaling pathway. Carcinogenesis 45 (6), 399–408. 10.1093/carcin/bgae008 38306794

[B36] Yunfang L. Yu H. Zhang C. Cheng Y. Hu L. Meng X. (2008). Protective effects of berberine on radiation-induced lung injury via intercellular adhesion molecular-1 and transforming growth factor-beta-1 in patients with lung cancer. Eur. J. Cancer 44 (16), 2425–2432. 10.1016/j.ejca.2008.07.040 18789680

[B37] Zaigang Z. Jiang X. Yi L. Li C. Wang H. Xiong W. (2024). Mitochondria energy metabolism depression as novel adjuvant to sensitize radiotherapy and inhibit radiation induced-pulmonary fibrosis. Adv. Sci. (Weinh) 11 (26), e2401394. 10.1002/advs.202401394 38715382 PMC11234447

[B38] Zhang J. Li W. Xue S. Gao P. Wang H. Chen H. (2024). Qishen granule attenuates doxorubicin-induced cardiotoxicity by protecting mitochondrial function and reducing oxidative stress through regulation of Sirtuin3. J. Ethnopharmacol. 319, 117134. 10.1016/j.jep.2023.117134 37714227

[B39] Zhangyang Q. Yang W. Xue B. Chen T. Lu X. Zhang R. (2024). ROS-mediated lysosomal membrane permeabilization and autophagy inhibition regulate bleomycin-induced cellular senescence. Autophagy 20 (9), 2000–2016. 10.1080/15548627.2024.2353548 38762757 PMC11346523

[B40] Zhuang L. Zhang L. Toma M. A. Li D. Bian X. Pastar I. (2022). Integrative small and long RNA omics analysis of human healing and nonhealing wounds discovers cooperating microRNAs as therapeutic targets. Elife 11 (0), e80322. 10.7554/eLife.80322 35942686 PMC9374442

